# Potential Association between Shift Work and Serologic Response to Hepatitis B Vaccination among Manufacturing Workers in Republic of Korea

**DOI:** 10.3390/vaccines12091041

**Published:** 2024-09-11

**Authors:** Si-Ho Kim, Chang-Ho Chae

**Affiliations:** 1 Division of Infectious Diseases, Samsung Changwon Hospital, Sungkyunkwan University, Changwon 51353, Republic of Korea; siho.kim@samsung.com; 2 Department of Occupational and Environmental Medicine, Samsung Changwon Hospital, Sungkyunkwan University School of Medicine, Changwon 51353, Republic of Korea

**Keywords:** Hepatitis B, Hepatitis B vaccines, shift work, immunogenicity, vaccine

## Abstract

(1) Background: Shift work can affect physical health and the immune system by altering the body’s circadian rhythms. This study investigated the factors associated with the hepatitis B virus (HBV) vaccination response in manufacturing workers, classified by whether they engaged in shift work or not. (2) Methods: This retrospective observational study was conducted among adults employed at two manufacturing companies. Those with negative initial hepatitis B surface antibody (HBsAb) levels before vaccination and who subsequently received a three-dose series of HBV vaccine were enrolled. Hepatitis B surface antibodies were examined for 3 years after the first dose. The endpoint of this study was the failure of a seroprotective anti-HB response after vaccination (HBsAb < 10 mIU/mL). Binary logistic regression models were used to analyze factors associated with response failures. (3) Results: Of the 1103 eligible subjects, 337 (30.6%) were shift workers. The failure rate was numerically higher in the shift workers (9.2%) than in the non-shift workers (7.9%), without statistical significance (*p* = 0.405). However, after adjustment with the binary logistic regression models, the shift workers had a statistically significantly higher rate of response failures than the non-shift workers (odds ratio 2.87; 95% confidence interval 1.64–5.05, *p* < 0.001), as did males, older workers, those with a low initial anti-HB titer, those with a vitamin D deficiency, and current smokers. (4) Conclusions: Our findings suggest a possible association between shift work and the serologic responses to HBV vaccination. Novel strategies for vaccination should be considered for shift workers.

## 1. Introduction

In contemporary society, a significant number of individuals engage in shift work, whether in public safety and health services or for economic reasons. Nighttime work disrupts circadian rhythms, potentially leading to various adverse health effects [1]. Physically, it increases the incidence of metabolic syndrome [2], cardiovascular disease [3,4], and digestive diseases such as indigestion, constipation, gastritis, duodenitis, and stomach ulcers [5,6], while diabetes and hypothyroidism due to hormonal changes have also been reported to be associated with shift work [7,8]. Systematic reviews and meta-analyses have also reported that shift work increases the incidence of various cancers [9,10]. Mentally, shift work has been reported to increase the incidence of fatigue and depression due to sleep disruption [11,12]. Sleep has been shown to prime immune cells and provide timing cues for the hematopoietic circadian clock [13]. Recently published studies have reported that shift workers have immunologic impairments, such as imbalanced levels of circulating monocytes, T lymphocytes, and immunologic biomarkers, which might result from sleep disturbances [14,15]. Reduced immune responses following vaccination in shift workers was reported in a previous study [16], but the clinical relevance and the mechanisms for this potential association remain unclear [17].

Hepatitis B virus (HBV) is a leading cause of chronic hepatitis and can lead to liver cirrhosis and hepatocellular carcinoma [18]. To prevent HBV transmission, vaccines have been developed, with second-generation DNA recombinant vaccines being widely used for universal immunization. [19]. The overall response rate to HBV vaccination in immunocompetent individuals is approximately 90–95%, but various factors, including sex, age, smoking, and vitamin D status, can influence the effectiveness of the vaccine [20,21]. Previous studies have evaluated the associations between shift work and responses to HBV vaccination; however, these studies reported conflicting results [22,23].

In the Republic of Korea, the prevalence of hepatitis B surface antigens (HBsAgs) in the general population was over 8% before the national vaccination program was initiated in 1995. Since the program’s implementation, the prevalence of HBsAgs has steadily declined, reaching 2% in 2019 [24]. The vaccination program, which mandates hepatitis B vaccination under the Korean Infectious Disease Prevention and Control Act [24,25], aims to immunize all newborns with a three-dose regimen of the hepatitis B vaccine (10 μg/0.5 mL per dose) provided free of charge. After the program’s launch, the coverage rate of HBV vaccination among infants has been reported to exceed 95% [24].

Our objective in this study was therefore to investigate factors associated with the serologic response after HBV vaccination in manufacturing workers in the Republic of Korea, including shift workers, at general manufacturing workplaces.

## 2. Methods

### 2.1. Study Site and Study Population

This retrospective observational study used data from routine screening and HBV vaccination programs at two companies: an electronic component manufacturing company in Busan, South Korea, which had an HBV vaccination program in place from 2011 to 2023, and a shipyard in Geoje, South Korea, which had an HBV vaccination program in place from 2015 to 2023. We included some of the patient cohort from our previous study in this current study [21]. The HBV vaccination programs at these two companies consisted of administering 1.0 mL of Euvax-B^®^ (produced by LG Chem, Seoul, Korea) on a set schedule to employees who tested negative for hepatitis B surface antibodies (Anti-HBs) at annual check-ups. Vaccinations were usually given between 10 am and 12 am. All employees also underwent annual check-ups, and all vaccinations and external examinations were performed by medical staff at Samsung Changwon Hospital. Anti-HB and vitamin D levels were measured using a Cobas 8000^®^ automated analyzer from Roche Diagnostics (Mannheim, Germany). Hepatitis B surface antibody (HBsAb) detection by the Elecsys Anti-HBs II system is reported to have 100% sensitivity and over 99% specificity by the manufacturer [26].

### 2.2. Study Eligibility Criteria and Outcomes

The inclusion criteria for this study were as follows: (1) absence of documented history of HBV infection or vaccination; (2) a negative hepatitis B surface antigen test and a hepatitis B surface antibody (less than 10 mIU/mL) during the pre-vaccination examination; (3) no prior documented history of HBV vaccination; and (4) completion of a three-dose HBV vaccination series within the study period. Exclusion criteria included: (1) workers with missing data (such as pre-vaccination anti-HB titer data), (2) foreign nationals, and (3) workers with diabetes or chronic kidney disease.

In this study, shift workers were alternately assigned to day shifts or night shifts in a sequential manner. Day shifts consisted of four consecutive 12-h shifts from 7 am to 7 pm, followed by two days off, while night shifts consisted of four consecutive 12-h shifts from 7 pm to 7 am, followed by two days off. The duration of employment and the type of work were also recorded. Body mass index, hemoglobin A1c, serum 25-OH vitamin D, serum alanine aminotransferase, and serum aspartate aminotransferase levels, and results of renal function tests to identify underlying disease were collected from the electronic medical records of the mandatory medical examination the year the first vaccine dose was administered. We also collected information on age and sex, smoking history, and shift work status at the time of the first dose. Considering that the Korean National HBV vaccination program began in 1995, we included birth year 1995 or later as a variable potentially associated with vaccine responses [24]. This approach is based on the assumption that individuals born in or after 1995 are more likely to have received the HBV vaccination, even if their anti-HB levels were later confirmed to be negative. Study outcome was failure of serologic response after HBV vaccination. Hepatitis B surface antibody levels were monitored for 3 years following the first dose. Participants who did not achieve a hepatitis B surface antibody level of 10 mIU/mL or higher during this period were classified as having failed to develop a seroprotective anti-HB response. We conducted an analysis of factors associated with serologic response failure in the entire study population, followed by a subgroup analysis of these factors specifically among shift workers.

### 2.3. Statistical Aanalysis

All data were analyzed using SPSS for Windows version 25.0 (SPSS Inc., Chicago, IL, USA). To compare baseline characteristics between groups, Student’s *t*-test or the Mann-Whitney test was used for continuous variables, while the chi-square test or Fisher’s exact test was employed for categorical variables. Factors associated with HBV response failure, including whether the worker engaged in shift work, were assessed using forward stepwise binary logistic regression models. Variables that showed statistical significance in the univariable analysis, as well as those deemed potentially clinically relevant, were included in the multivariable analysis. A *p* value of less than 0.05 was considered statistically significant.

### 2.4. Ethical Considerations

The study received approval from the Institutional Review Board of Samsung Medical Center (IRB file number: SCMC 2024-04-019) with a waiver of informed consent. The waiver was granted because the study was an observational retrospective analysis, and all patient data were anonymized prior to analysis.

## 3. Results

### 3.1. Study Population

**Figure 1:**
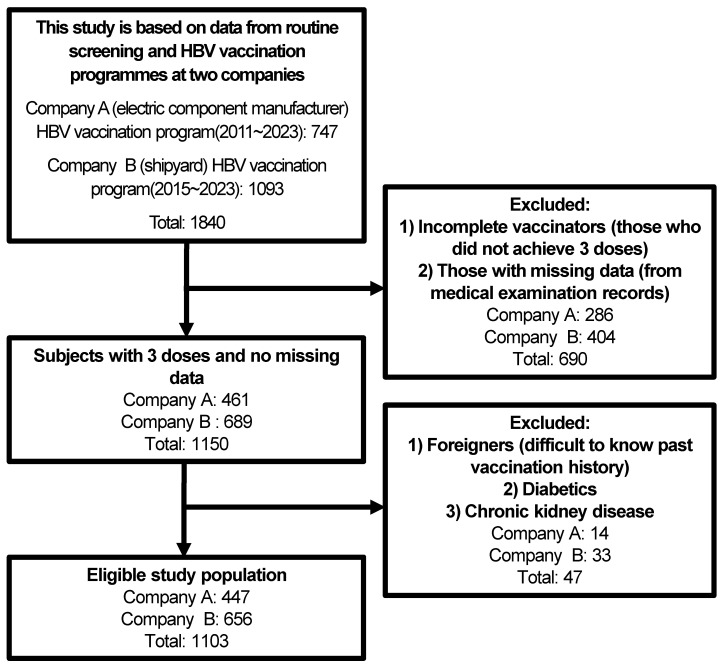
Flow chart of participant selection.

A total of 1103 workers met the study inclusion criteria (Figure 1).

Of the study population, 337 (30.6%) were shift workers and 766 (69.4%) were non-shift workers. There were statistical differences between the shift and non-shift workers in age (25.6 ± 4.3 vs. 31.7 ± 6.3 years, respectively, *p* < 0.001), female gender (44.2% vs. 12.1%, respectively, *p* < 0.001), body mass index (23.3 ± 3.8 vs. 24.6 ± 3.5 kg/m^2^, respectively, *p* < 0.001), HBsAb titer prior to vaccination (3.53 ± 2.19 vs. 4.24 ± 2.29 ng/mL, respectively, *p* < 0.001), and serum 25-OH vitamin D levels (13.7 ± 5.5 vs. 16.8 ± 6.7 ng/mL, respectively, *p* < 0.001). When defining vitamin D deficiency as a 25-OH vitamin D level below 20 ng/mL, approximately 90% of the shift workers had a vitamin D deficiency, compared to about 65% of the non-shift workers. Current smoking status, and the mean values of HbA1C, serum creatinine, ALT, and AST were not statistically different between the shift workers and non-shift workers (Table 1).

### 3.2. Immune Response after HBV Vaccination

There were 2516 HBsAb measurements (mean 2.3 per worker) conducted over three years following the three-dose series of HBV vaccination. The number of days until the determination of the serologic response after the third vaccination—calculated from the date of the first examination for the patients with a negative antibody response, and from the date of the first positive result for the patients with a positive antibody response—had a median of 147 days (interquartile range 53–218 days).

Ninety (8.2%) workers were classified as non-responders. The failure rate was slightly higher in the shift workers (9.2%) than in the non-shift workers (7.9%), without statistical significance (*p* = 0.405). The mean age of the non-responders (32.3 ± 6.5 years) was statistically higher than the mean age of the responders (29.6 ± 6.4 years, *p* < 0.001). In addition, the non-responders were more likely to be male, current smokers, have lower blood 25-OH vitamin D levels, and have significantly lower pre-vaccination anti-HB titers than the responders. There is no association in the serologic responses between employees who were born in 1995 or later and those who were not (Table 2).

### 3.3. Factors Associated with HBV Vaccination Response Failure

In the binary regression models, the shift workers had a higher risk of response failure than the non-shift workers (adjusted OR 2.87, 95% CI 1.64–5.05, *p* < 0.001). In addition, older age (adjusted OR 1.06, 95% CI 1.02–1.10 per 1-year, *p* = 0.006), male gender (adjusted OR 3.47, 95% CI 1.39–8.69, *p* = 0.008), vitamin D deficiency (<20 ng/mL) (adjusted OR 2.57, 95% CI 1.31–5.07, *p* = 0.006), and current smoking (adjusted OR 1.64, 95% CI 1.02–2.62, *p* = 0.040) were also associated with a response failure (Table 3). Conversely, a higher pre-vaccination HBsAb titer (adjusted OR 0.44 per 1 mIU/mL, 95% CI 0.33–0.60, *p* < 0.001) was associated with a lower risk of a response failure (Table 3).

### 3.4. Subgroup Analysis of Factors Associated with HBV Response Failure Specifically among Shift and Non-Shift Workers

In the subgroup analysis among the shift workers, the shift workers who did not respond to the vaccine were older than those who did (25.3 ± 4.2 vs. 28.3 ± 4.4 years, respectively, *p* < 0.001). Additionally, employment duration, male sex, HBsAb titer prior to vaccination, and serum creatinine levels showed statistically significant differences between the responders and non-responders. In the subgroup analysis among the shift workers, age, employment duration, male sex, smoking status, anti-HBs titer prior to vaccination, serum 25-OH vitamin D levels, and serum creatinine levels showed statistically significant differences between the responders and non-responders (Table 4).

In the binary regression models for the shift workers, employment duration (OR 1.14, 95% CI 1.03–1.25, *p* = 0.010) and male sex (OR 2.74, 95% CI 1.05–7.13, *p* = 0.039) were independently associated with a HBV vaccine response failure. Conversely, a higher pre-vaccination anti-HB titer (adjusted OR 0.59 per 1 mIU/mL, 95% CI 0.43–0.80, *p* < 0.001) was associated with a lower risk of a response failure (Table 5). In the binary regression models for the non-shift workers, employment duration was not included in the final model. Age, male sex, vitamin D deficiency, and current smoking status were associated with an increased risk of a vaccine response failure. Conversely, a higher pre-vaccination anti-HB titer was associated with a lower risk of a response failure, as shown in the subgroup multivariable analysis for the shift workers (Table 6).

## 4. Discussion

Our study suggests that shift work may be associated with lower serologic responses after HBV vaccination, alongside known clinical factors such as old age, male gender, current smoking, a lower pre-vaccination HBsAb titer, and a low vitamin D level.

Several studies have evaluated the impact of shift work on vaccine responses [16,22,23]. A key mechanism for reducing the effectiveness of vaccinations in shift workers, including the HBV vaccine, is thought to be disrupted circadian rhythms and chronic sleep problems, which impair the body’s ability to mount an effective immune response [27]. In addition, shift work is associated with higher rates of heart disease, diabetes, and other conditions, which can lead to the increased production of stress hormones such as cortisol, which can suppress immune function and reduce the efficacy of vaccines [17,28]. However, the results of studies have been inconsistent [16,22,23]. A previous study on meningococcal C meningitis vaccines showed that night workers exhibited a weak humoral response, which was associated with a decrease in total sleep duration and an alteration of circadian rhythm [16]. Two prior studies have evaluated the association between HBV vaccination and shift work [22,23]. One study reported that shift workers among health care providers showed a decreased protective antibody response compared to non-shift workers (76.5% vs. 96.5%, *p* = 0.001). However, this study did not assess if other characteristics differed between the shift and non-shift workers, and did not conduct statistical adjustments for other clinical factors [23]. Another study reported no association between night shift work among health care providers and a seroprotective response after HBV vaccination after adjusting for other clinical factors [22]. Our study is the first to demonstrate a robust association between shift work and serologic responses to HBV vaccination among non-healthcare providers. In this study, there were no significant differences in response failures between the shift and non-shift workers in the univariate analysis. However, in the multivariate analysis that was adjusted for other clinical factors, shift work was associated with a response failure to HBV vaccination. The difference between the univariate and multivariate analysis may be due to the younger age and higher proportion of women among the shift workers in this study, which are favorable factors for antibody responses after HBV vaccination [29,30,31]. Therefore, when studying the effect of shift work on the immune response to vaccination, not only the type of shift work but also the gender and age of the shift workers should be considered. Moreover, it is difficult to provide a clear explanation for the conflicting results between the current study and our study; however, this might be due to various types of shift work, different hours and regularity, and differing degrees of circadian disruption and sleep disturbance [32].

In this study, blood vitamin D levels were significantly lower in the shift workers than the non-shift workers (Table 1). In addition, the HBV vaccine non-response rate was higher in the vitamin D-deficient group. No significant difference in vitamin D deficiency rates was observed between the non-responders and responders to the HBV vaccine among the shift workers. However, given that over 90% of the shift workers were vitamin D deficient, this may have limited the ability to detect a statistically significant difference. Although more research is needed to understand the effects of vitamin D on B-cell-mediated humoral immunity, it has been hypothesized that the role of vitamin D in humoral immunity is related to its immune homeostasis and immunomodulatory effects [33,34]. Several studies have suggested the possibility of a lower serologic response to vaccination among populations with vitamin D deficiency, or a higher serologic response after vitamin D supplementation [21,33]. There was a study that determined that the coadministration of 1,25-Dihydroxyvitamin D3 and an inactive polio vaccine could enhance serum immunoglobulin G and salivary immunoglobulin M [35]. Although it has been debated, several human studies have suggested a lower immunologic response to HBV vaccinations among individuals with vitamin D deficiency [21,36]. Consistent with these findings, our study suggests that vitamin D deficiency is associated with a reduced serologic response, and the tendency for lower serum vitamin D levels among shift workers could contribute to a diminished serologic response following vaccination.

Among shift workers, the serologic responses after vaccination were associated with male sex and pre-vaccination HBsAb titer. These results were consistent with the findings from the entire study population. In addition, employment duration was an independent factor associated with response failures. Given that employment duration was not a dependent factor for response failures among non-shift workers, this suggests that shift work itself may be associated with the failure of a serologic response to the HBV vaccine, potentially exacerbated by the profound vitamin D deficiency observed in the shift worker group. Therefore, strategies to enhance the HBV vaccine response in shift workers might prioritize addressing the prolonged employment duration and aim to reduce the vitamin D deficiency seen within this population.

This study had several limitations. First, being a retrospective observational study, it was subject to the inherent limitations of this study type, namely selection bias and information bias. Second, certain variables that might affect vaccine responses, such as work position and shift mode at the time of vaccination, could not be collected. We assumed that regular workers typically do not have an immunodeficiency; however, we did not evaluate all factors related to immunodeficiencies during the routine medical examinations. In addition, because it is difficult to obtain documented vaccination history from childhood, there is still the possibility that some of the study population had been vaccinated before. Although there is no association in the serologic responses between employees born in 1995 or later and those born before, and it could be suggested that these individuals were not vaccinated prior to study participation, this cannot be considered direct evidence due to the small sample size (3.5% of the total study population). Third, we only evaluated serologic responses after HBV vaccination. Further research is needed to evaluate the association between T-cell responses and factors such as shift work following HBV vaccination [37,38]. Fourth, we did not measure the Hepatitis B core antibody (HBcAb). Individuals with isolated HBcAb may have previously experienced a decline in HBsAb levels over time and could respond better to a new challenge (booster) through vaccination. However, based on epidemiological data from the Republic of Korea, the prevalence of isolated HBcAb is 1% among individuals aged 21–40 and 4.9% among those aged 41–50 [39]. Therefore, we assumed that the impact would be minimal, considering the age of our study population. Despite these limitations, we performed a large-scale investigation of the response of shift workers in general manufacturing to HBV vaccination, whereas most previous studies on the HBV immune response and shift work have focused on healthcare providers. Additionally, we analyzed the relationships between other clinical factors and a serologic response after HBV vaccination in addition to the relationship between shift work and serologic responses.

## 5. Conclusions

Our findings suggest that shift work may be associated with a failure to achieve a seroprotective response after HBV vaccination in young workers in a general manufacturing workplace. Additionally, our results are consistent with previous studies indicating that older age, male gender, vitamin D deficiency, and current smoking are associated with a lower seroprotective response. Therefore, HBV vaccination strategies for shift workers should be carefully planned, taking into account potential circadian rhythm disruptions and other clinical factors known to affect the body’s response to vaccination.

## Figures and Tables

**Table 1:** General and clinical characteristics of participants by shift work status at time of vaccination.

Variables		Total (n = 1103)	Shift Work		*p*-Value
			No (n = 766)	Yes (n = 337)	
Age (years) ^†^		29.9 ± 6.5	31.7 ± 6.3	25.6 ± 4.3	<0.001
Birth year 1995 or later		39 (3.5)	15 (2.0)	24 (7.1)	<0.001
Age group	≤29	625 (56.7)	337 (44.0)	288 (85.5)	<0.001
	30~39	366 (33.2)	319 (41.6)	47 (13.9)	
	≥40	112 (10.2)	110 (14.4)	2 (0.6)	
Sex	Male	861 (78.1)	673 (87.9)	188 (55.8)	<0.001
	Female	242 (21.9)	93 (12.1)	149 (44.2)	
Work place	Company A	447 (40.5)	110 (14.4)	337 (100)	<0.001
	Company B	656 (59.5)	656 (85.6)	0 (0)	
Type of work	Production department	645 (58.5)	331 (43.2)	314 (93.2)	<0.001
	Administrative department	391 (35.4)	381 (49.7)	10 (3.0)	
	Support department	67 (6.1)	54 (7.0)	13 (3.9)	
Employment duration (years)		6.58 ± 5.63	7.33 ± 6.18	4.91 ± 3.62	<0.001
Smoking	Current smoker	405 (36.7)	278 (36.3)	127 (37.7)	0.658
	None or ex-smoker	698 (63.3)	488 (63.7)	210 (62.3)	
Body mass index (kg/m^2^)		24.2 ± 3. 7	24.6 ± 3.5	23.3 ± 3.8	<0.001
Anti-HB titer prior to vaccination (mIU/mL)		3.75 ± 2.24	3.53 ± 2.19	4.24 ± 2.29	<0.001
Serum 25-OH vitamin D (ng/mL)		15.9 ± 6.5	16.8 ± 6.7	13.7 ± 5.5	<0.001
Vitamin D deficiency (25-OH vitamin D level below 20 ng/mL)		860 (78.0)	667 (64.8)	303 (89.9)	<0.001
Hemoglobin A1c (mg/dL)		5.34 ± 0.3	5.33 ± 0.3	5.36 ± 0.3	0.082
Serum creatinine (mg/dL)		0.96 ± 0.12	0.96 ± 0.13	0.96 ± 0.1	0.284
AST (IU/L)		23.7 ± 13.96	23.7 ± 16.20	23.8 ± 6.41	0.895
ALT (IU/L)		26.8 ± 16.92	26.8 ± 19.27	26.8 ± 9.67	0.967

^†^ Continuous variables are expressed as mean ± SD, while categorical variables are expressed as numbers (%).

**Table 2:** Differences in variables according to post-vaccination serologic responses.

Variables			HBs IgG Levels (Post-Vaccination)		*p*-Value
			≥10 mIU/mL Responder (n = 1013)	<10 mIU/mL Non-Responder (n = 90)	
Shift work		Yes	306 (30.2)	31 (34.4)	0.405
		No	707 (69.8)	59 (65.6)	
Type of work		Production department	591 (58.3)	54 (60.0)	0.858
		Administrative department	361 (35.6)	30 (33.3)	
		Support department	61 (6.0)	6 (6.7)	
Employment duration (years)			6.43 ± 5.58	8.42 ± 6.00	<0.001
Age (years) ^†^			29.6 ± 6.4	32.3 ± 6.5	<0.001
Birth year 1995 or later	Yes		36 (3.6)	3 (3.3)	>0.999
	No		977 (96.4)	87 (96.7)	
Age group	≤29		594 (58.6)	31 (34.4)	<0.001
	30~39		323 (31.9)	43 (47.8)	
	≥40		96 (9.5)	16 (17.8)	
Sex	Male		777 (76.7)	84 (93.3)	<0.001
	Female		236 (23.3)	6 (6.7)	
Smoking	Current smoker		324 (32.0)	45 (50.0)	0.001
	None or ex-smoker		689 (68.0)	45 (50.0)	
Body mass index (kg/m^2^)			24.1 ± 3.7	24.7 ± 3.7	0.181
Anti-HB titer prior to vaccination			3.9 ± 2.3	2.3 ± 1.0	<0.001
Serum 25-OH vitamin D (ng/mL)			16.0 ± 6.6	14.5 ± 4.6	0.004
Vitamin D deficiency (25-OH vitamin D level below 20 ng/mL)			781 (77.1)	79 (87.8)	0.019
Hemoglobin A1c (mg/dL)			5.35 ± 0.29	5.30 ± 0.31	0.136
Serum creatinine (mg/dL)			0.96 ± 0.12	0.97 ± 0.12	0.407
AST (IU/L)			23.8 ± 14.3	23.2 ± 9.4	0.467
ALT (IU/L)			26.7 ± 17.1	29.4 ± 15.4	0.208

^†^ Continuous variables are expressed as mean ± SD, while categorical variables are expressed as numbers (%).

**Table 3:** Factors associated with HBV vaccination response failures.

Variables	Univariate		Binary Logistic Model		
	OR	95% CI	OR	95% CI	*p*-Value
Shift work	1.21	0.77–1.91	2.87	1.64–5.05	<0.001
Employment duration (per year)	1.06	1.02–1.09			
Age (per year)	1.06	1.03–1.09	1.06	1.02–1.10	0.006
Sex (male)	4.25	1.83–9.79	3.47	1.39–8.69	0.008
HBsAb titer prior to vaccination (per 1 mIU/mL)	0.45	0.34–0.61	0.44	0.33–0.60	<0.001
Current smoking	2.12	1.38–3.28	1.64	1.02–2.62	0.040
Vitamin D deficiency (25-OH vitamin D level below 20 ng/mL)	2.13	1.12–4.08	2.57	1.31–5.07	0.006

The values presented in the table were included in the multivariable analysis.

**Table 4:** Differences in variables according to post-vaccination serologic responses among shift workers.

Variables		Shift Workers			Non Shift Workers		
		HBs IgG Levels (Post-Vaccination)		*p*-Values	HBs IgG Levels (Post-Vaccination)		*p*-Value
		≥10 mIU/mL Responder (n = 306)	<10 mIU/mL Non-Responder (n = 31)		≥10 mIU/mL Responder (n = 707)	<10 mIU/mL Non-Responder (n = 59)	
Type of work	Production department	285 (93.1)	29 (93.5)	>0.999	306 (43.3)	25 (42.4)	0.905
	Administrative department	9 (2.9)	1 (3.2)		352 (49.8)	29 (49.2)	
	Support department	12 (3.9)	1 (3.2)		49 (6.9)	5 (9.3)	
Employment duration (year)		4.69 ± 3.48	7.10 ± 4.29	0.001	7.18 ± 6.12	9.12 ± 6.65	0.022
Age (years) ^†^		25.3 ± 4.2	28.3 ± 4.4	<0.001	31.5 ± 6.3	34.4 ± 6.5	<0.001
Birth year 1995 or later	Yes	22 (7.2)	2 (6.5)	>0.999	14 (2.0)	1 (1.7)	>0.999
	No	284 (92.8)	29 (93.5)		693 (98.0)	58 (98.3)	
Age group	≤29	269(87.9)	19(61.3)	0.001	325 (46.9)	12 (20.3)	<0.001
	30~39	35 (11.4)	12 (38.7)		288 (40.7)	31 (52.5)	
	≥40	2 (0.7)	0 (0.0)		94 (13.3)	16 (27.1)	
Sex	Male	163 (53.3)	25 (19.4)	0.003	614 (86.8)	59 (100.0)	0.001
	Female	143 (46.7)	6 (6.7)		93 (13.2)	0 (0.0)	
Smoking	Current smoker	96(31.4)	15 (48.4)	0.055	228 (32.2)	30 (50.8)	0.004
	None or ex-smoker	210 (68.6)	16 (51.6)		479 (67.8)	29 (49.2)	
Body mass index (kg/m^2^)		23.2 ± 3.8	24.2 ± 3.4	0.182	24.5 ± 3.5	24.9 ± 3.8	0.661
HBsAb titer prior to vaccination		4.4 ± 2.3	2.6 ± 1.6	<0.001	3.7 ± 2.2	2.1 ± 0.2	<0.001
Serum 25-OH vitamin D (ng/mL)		13.7 ± 5.6	13.8 ± 3.8	0.948	17.0 ± 6.7	14.8 ± 5.0	0.013
Vitamin D deficiency (25-OH vitamin D level below 20 ng/mL)		273 (90.1)	30 (96.8)	0.342	508 (71.9)	49 (83.1)	0.064
Hemoglobin A1c (mg/dL)		5.28 ± 0.26	5.35 ± 0.40	0.341	5.39 ± 0.30	5.28 ± 0.26	0.237
Serum creatinine (mg/dL)		0.97 ± 0.10	0.94 ± 0.11	0.028	0.95 ± 0.13	0.99 ± 0.12	0.044
AST (IU/L)		23.8 ± 6.1	24.0 ± 9.1	0.158	23.8 ± 16.6	22.9 ± 9.6	0.728
ALT (IU/L)		26.6 ± 9.1	28.7 ± 14.5	0.683	26.7 ± 19.5	28.3 ± 15.9	0.168

^†^ Continuous variables are expressed as mean ± SD, while categorical variables are expressed as numbers (%).

**Table 5:** Factors associated with HBV vaccine response failures among shift workers.

Variables	Univariate		Binary Logistic Model		
	OR	95% CI	OR	95% CI	*p*-Value
Age (per year)	1.16	1.07–1.25			
Employment duration (per year)	1.16	1.06–1.26	1.14	1.03–1.25	0.010
Sex (male)	3.66	1.46–9.16	2.74	1.05–7.13	0.039
HBsAb titer prior to vaccination (per 1 mIU/mL)	0.56	0.41–0.77	0.59	0.43–0.80	0.001
Current smoking	2.05	0.97–4.32			
Serum creatinine (per 1 mg/dL)	0.07	0.00–1.58			

The values presented in the table were included in the multivariable analysis.

**Table 6:** Factors associated with HBV vaccine response failures among non-shift workers.

Variables	Univariate		Binary Logistic Model		
	OR	95% CI	OR	95% CI	*p*-Value
Age (per year)	1.07	1.03–1.11	1.05	1.00–1.09	0.035
Employment duration (per year)	1.05	1.01–1.09			
HBsAb titer prior to vaccination (per 1 mIU/mL)	0.19	0.07–0.52	0.17	0.06–0.48	0.001
Vitamin D deficiency (25-OH vitamin D level below 20 ng/mL)	1.92	0.95–3.86	2.50	1.19–5.22	0.015
Current smoking	2.17	1.27–3.71	2.04	1.14–3.65	0.017
Serum creatinine (per 1 mg/dL)	11.4	1.35–95.56	23.07	2.07–256.67	0.011

The values presented in the table were included in the multivariable analysis. Sex was not included in the multivariable model because there were no non-responder events among the females.

## Data Availability

The raw data supporting the conclusions of this article will be made available by the authors on request.
